# Large Spin Coherence Length and High Photovoltaic Efficiency of the Room Temperature Ferrimagnet Ca_2_FeOsO_6_ by Strain Engineering

**DOI:** 10.1002/advs.202106037

**Published:** 2022-07-21

**Authors:** Paresh C. Rout, Udo Schwingenschlögl

**Affiliations:** ^1^ Physical Sciences and Engineering Division (PSE) King Abdullah University of Science and Technology (KAUST) Thuwal 23955‐6900 Saudi Arabia

**Keywords:** double perovskite, ferrimagnetism, photovoltaics, spin coherence, strain engineering

## Abstract

The influence of epitaxial strain on the electronic, magnetic, and optical properties of the distorted double perovskite Ca_2_FeOsO_6_ is studied. These calculations show that the compound realizes a monoclinic structure with *P*2_1_/*n* space group from −6% to +6% strain. While it retains ferrimagnetic ordering with a net magnetic moment of 2 μ_B_ per formula unit at low strain, it undergoes transitions into E‐antiferromagnetic and C‐antiferromagnetic phases at −5% and +5% strain, respectively. It is shown that spin frustration reduces the critical temperature of the ferrimagnetic ordering from the mean field value of 600–350 K, in excellent agreement with the experimental value of 320 K. It is also shown that the critical temperature can be tuned efficiently through strain and that the spin coherence length surpasses that of Sr_2_FeMoO_6_ under tensile strain. An indirect‐to‐direct bandgap transition is observed at +5% strain. Localization of the valence and conduction states on different transition metal sublattices enables efficient electron–hole separation upon photoexcitation. The calculated spectroscopic limited maximum efficiency of up to 33% points to excellent potential of Ca_2_FeOsO_6_ in solar cell applications.

## Graphical abstract

Spin frustration is found to reduce the critical temperature of the ferrimagnetic ordering of Ca_2_FeOsO_6_ to reconcile a discrepancy between experiment and theory. The spin coherence length surpasses that of Sr_2_FeMoO_6_ under tensile strain. Localization of the valence and conduction states on different transition metal sublattices enables efficient electron–hole separation upon photoexcitation.

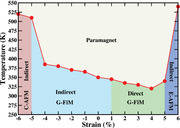

## 1 Introduction

Double perovskites with the general formula A_2_BB′O_6_, formed by corner‐sharing BO_6_ and B'O_6_ octahedra and 12‐coordinated rare‐earth or alkaline‐earth A cations, draw the attention of condensed matter physicists, solid state chemists, and material scientists due to the possibility of having different transition metal ions at the B and B' sites, providing additional degrees of freedom over standard perovskites (ABO_3_).^[^ ^1^ ^]^ Double perovskites are known to show diverse electronic and magnetic properties^[^ ^2^ ^]^ with potential in the fields of spintronics,^[^ ^3^ ^]^ multiferroicity,^[^ ^4^ ^]^ magnetocapacitivity,^[^ ^5^ ^]^ and solar energy harvesting.^[^ ^6^ ^]^ Particularly double perovskites with 3d B and 4d/5d B' ions have gained great attention due to the ferrimagnetic (FiM) half‐metallic natures of Sr_2_FeMoO_6_^[^ ^3^, ^7^, ^8^ ^]^ and Sr_2_FeReO_6_^[^ ^9^, ^10^, ^11^ ^]^ with giant tunneling magnetoresistance above room temperature. Various candidates for spintronic applications at elevated temperatures have been explored.^[^ ^12^, ^13^, ^14^ ^]^ For example, the double perovskite Sr_2_CrOsO_6_ realizes a FiM phase below 725 K,^[^ ^13^ ^]^ the highest reported critical temperature in the class of perovskites and double perovskites.^[^ ^12^ ^]^ Similarly, Sr_2_FeOsO_6_ is known for its lattice instability and competing magnetic interactions.^[^ ^15^ ^]^ The double perovskite Ca_2_FeOsO_6_, which recently has been synthesized under high temperature and pressure, adopts a monoclinic *P*2_1_/*n* structure and is reported to be a G‐FiM semiconductor with a high critical temperature of 320 K.^[^ ^16^ ^]^ However, the value of 600 K predicted theoretically in the mean‐field approximation with nearest‐neighbor (NN) couplings is much higher,^[^ ^17^ ^]^ which lacks an explanation. In addition, it is not clear why the critical temperature is much lower than in the case of closely related Sr_2_CrOsO_6_. FiM materials are preferable over ferromagnetic (FM) and antiferromagnetic (AFM) materials for spintronic applications, as they can simultaneously provide a large spin coherence length and a bulk‐like torque due to their net magnetic moment.^[^ ^18^, ^19^ ^]^

We solve the issue of the overestimated critical temperature of G‐FiM Ca_2_FeOsO_6_ by computing the magnetic coupling constants based on total energies and an effective Heisenberg model.^[^ ^20^ ^]^ Monte Carlo simulations show that spin frustration significantly reduces the critical temperature. Since strain can induce tunable functionalities in transition metal oxides,^[^ ^21^, ^22^, ^23^ ^]^ particularly in highly distorted double perovskites,^[^ ^24^ ^]^ we investigate the effect of epitaxial strain on the electronic, magnetic, and optical properties. We show that the monoclinic *P*2_1_/*n* structure and G‐FiM ordering are retained up to ±4% strain (with the positive/negative sign representing tensile/compressive strain). Suppression of the spin frustration turns out to enhance the critical temperature, providing access to a magnetic semiconductor far above room temperature. We discover that the spin coherence length under tensile strain exceeds that of the famous double perovskite spintronic material Sr_2_FeMoO_6_,^[^ ^3^ ^]^ demonstrating exceptional potential of Ca_2_FeOsO_6_ in room temperature spintronic applications. Moreover, we show that Ca_2_FeOsO_6_ undergoes an indirect‐to‐direct bandgap transition at +1% strain, making it interesting for photovoltaic applications due to the possibility of electron–hole separation between the Fe and Os sublattices and, consequently, low electron–hole recombination. Indeed, we obtain at +1% strain a spectroscopic limited maximum efficiency (SLME) of 33%.

## 2 Computational Details

We adopt spin‐polarized density functional theory^[^ ^25^ ^]^ in the generalized gradient approximation (Perdew–Burke–Ernzerhof) of the exchange correlation potential. The electronic correlation effects in the 3d and 5d transition metal orbitals are addressed by considering an onsite interaction,^[^ ^26^ ^]^ for which we adopt the established literature values of 5 eV for Fe and 2 eV for Os.^[^ ^17^ ^]^ Despite the presence of heavy elements, spin‐orbit coupling turns out to have no relevant impact on the electronic structure of Ca_2_FeOsO_6_ without strain, in agreement with ref. [17], except for a slight increase of the atomic magnetic moments without affecting the net magnetic moment, and therefore is neglected in the calculations under strain to reduce the computational costs. A plane wave cutoff energy of 90 Ry is used in the wave function expansion and a cutoff energy of 640 Ry for the augmentation charge. The Brillouin zone is integrated on an 8 × 8 × 6 Monkhorst–Pack *k*‐mesh in the structure optimization, which we find to provide convergence of the total energy, and on a 14 × 14 × 12 Monkhorst–Pack *k*‐mesh in the calculation of the density of states. The total energy convergence criterion is set to 10^−8^ Ry and structures are optimized until the Hellmann–Feynman forces stay below 10^−5^ Ry Bohr^−1^.

**Figure 1:**
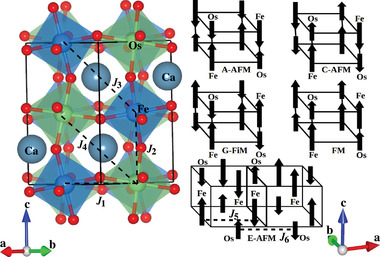
Exchange paths connecting the Fe and Os atoms and the considered magnetic orderings.

Employing this methodology, we obtain for bulk Ca_2_FeOsO_6_ the lattice parameters *a* = 5.43 Å, *b* = 5.58 Å, and *c* = 5.73 Å (which agree well with the experimental values of *a* = 5.39 Å, *b* = 5.51 Å, and *c* = 5.68 Å^[^ ^16^ ^]^), G‐FiM ordering, and a semiconducting state with indirect band gap. We then consider a 20‐atom $\sqrt{2}\times \sqrt{2}\times 2$ tetragonal supercell of the pseudocubic perovskite structure with rock salt ordering of the Fe and Os ions, see **Figure** **1**, for both *P*2_1_/*n* and *I*4/*m* symmetries. Starting from the pseudocubic lattice parameter of 3.90 Å, we mimic epitaxial strain ϵ by varying the in‐plane lattice constant as $a\left(\varepsilon \right)=\sqrt{2}\left(1+\varepsilon \right)\times 3.90$ Å, limited to values that realistically can be achieved by a substrate. The length and angle of the out‐of‐plane lattice vector are optimized for each strain value simultaneously with the atomic positions, representing (001) epitaxial growth. This procedure is executed for different magnetic orderings, see Figure 1, and for both the *P*2_1_/*n* and *I*4/*m* symmetries to capture the strain effect on the relative energies of the magnetic phases. The absorption spectra are calculated for the energetically favorable configurations as α(ω)=2ωcRe[ε(ω)]2+Im[ε(ω)]2−Re[ε(ω)], where ω is the frequency, *c* is the speed of light, and ϵ(ω) is the dielectric function.^[^ ^27^ ^]^

## 3 Results and Discussion

**Figure 2:**
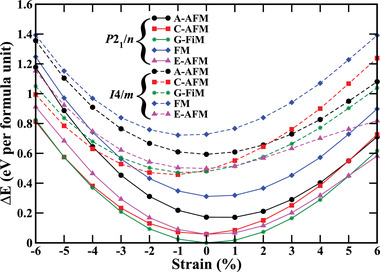
Relative energies (with respect to the global minimum) of the magnetic orderings under strain for the *P*2_1_/*n* (solid lines) and *I*4/*m* (dashed lines) symmetries.

We show in **Figure** **2** the total energies obtained for the *P*2_1_/*n* and *I*4/*m* symmetries with respect to the global minimum, demonstrating that the *P*2_1_/*n* symmetry is favorable in the considered range of strain. Within the *P*2_1_/*n* symmetry the G‐FiM ordering is favorable from −4% to 4% strain and magnetic phase transitions into C‐AFM and E‐AFM phases are encountered at −5% and +5% strain, respectively. The G‐FiM ordering leads to a net magnetic moment of 2 μ_B_ per formula unit, while the net magnetic moment is zero for both the C‐AFM and E‐AFM orderings due to exact cancellation of the atomic magnetic moments. While the G‐FiM and C‐AFM phases stay very close in energy for increasing compressive strain, the phase transition is related to increasing Fe−O−Os bond angles along the *c*‐axis, which favor FM superexchange with the in‐plane coupling remaining AFM due to persistent buckling. The G‐FiM to E‐AFM phase transition can be attributed to enhanced in‐plane Fe−Fe and Os−Os couplings combined with reduced in‐plane Fe−Os couplings (see below).

To gain more insight into the magnetic phases and to understand the evolution of their critical temperatures under strain, we next extract the magnetic coupling constants using the Heisenberg spin Hamiltonian $H=-\sum_{i,j}J_{i,j}\vec{S_{i}}\cdot \vec{S_{j}}$, where *J*_*i*, *j*_ is the coupling constant between sites *i* and *j*, and $\vec{S_{i}}$ and $\vec{S_{j}}$ are the spin vectors.^[^ ^28^ ^]^ We consider the in‐plane NN coupling (*J*_1_), the out‐of‐plane NN coupling (*J*_2_), and the next‐nearest‐neighbor (NNN) couplings (*J*_3_, *J*_4_), see Figure 1. For the E‐AFM ordering we also consider the Fe−Fe (*J*_5_) and Os−Os (*J*_6_) couplings along the *a*‐axis. When we assume that the spin vectors are collinear with $|\vec{S_{i}}|=1$, as the real magnitude later will be taken into account in the Monte Carlo simulations, we can compute the magnetic coupling constants by solving the coupled equations *E*_1_ = *E*_0_ + 8*J*_1_ − 4*J*_2_ − 8*J*_3_ − 8*J*_4_ (A‐AFM), *E*_2_ = *E*_0_ − 8*J*_1_ + 4*J*_2_ − 8*J*_3_ − 8*J*_4_ (C‐AFM), *E*_3_ = *E*_0_ − 8*J*_1_ − 4*J*_2_ + 8*J*_3_ + 8*J*_4_ (G‐FiM), *E*_4_ = *E*_0_ + 8*J*_1_ + 4*J*_2_ + 8*J*_3_ + 8*J*_4_ (FM), *E*_5_ = *E*_0_ + 8*J*_3_ − 8*J*_4_ (G‐FiM with one Os spin flipped), *E*_6_ = *E*_0_ − 8*J*_3_ + 8*J*_4_ (G‐FiM with one Fe spin flipped), *E*_7_ = *E*_0_ − 4*J*_2_ − 4*J*_5_ + 4*J*_6_ (E‐AFM), *E*_8_ = *E*_0_ − 4*J*_2_ − 4*J*_5_ + 4*J*_6_ (E‐AFM with FM coupling along the *c*‐axis), *E*_9_ = *E*_0_ − 4*J*_2_ − 4*J*_5_ + 4*J*_6_ (E‐AFM rotated by 90° around the *c*‐axis), and *E*_10_ = *E*_0_ − 4*J*_2_ − 4*J*_5_ + 4*J*_6_ (E‐AFM rotated by 90° around the *c*‐axis with FM coupling along the *c*‐axis), where *E*_0_ is the lattice energy and *E*_1_ to *E*_10_ are the total energies of the magnetic orderings obtained from density functional theory.

**Figure 3:**
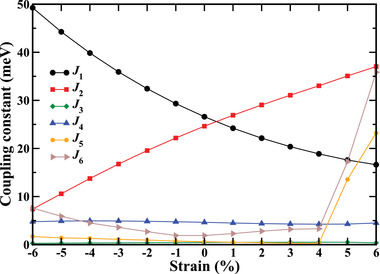
Variation of the magnetic coupling constants under strain.

**Figure 4:**
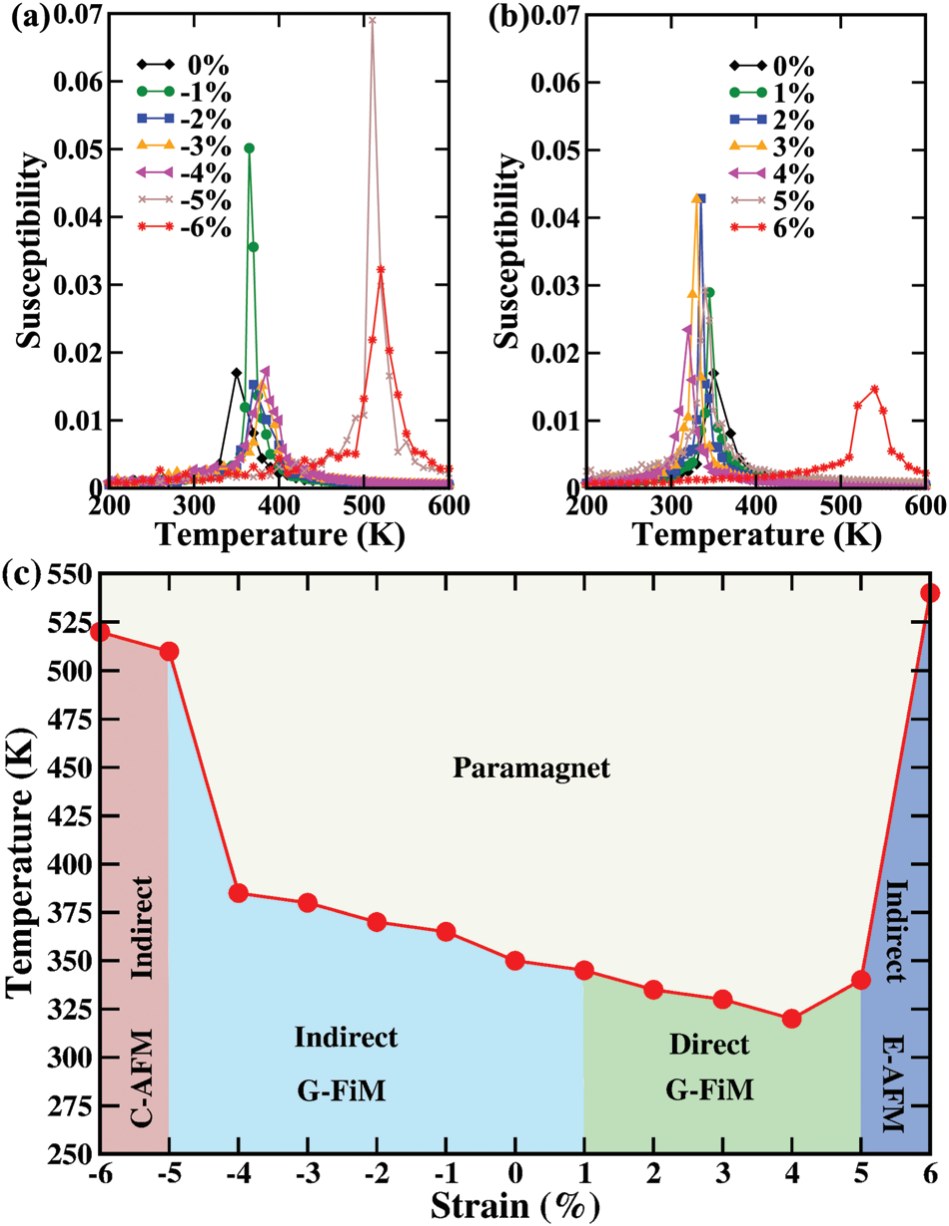
a,b) Temperature dependence of the magnetic susceptibilities of the ground state magnetic orderings at given strain when both the NN and NNN couplings are considered. The peaks mark the critical temperatures. c) Strain‐temperature phase diagram obtained from Monte Carlo simulations.

As can be seen in **Figure** **3**, both *J*_1_ and *J*_2_ are positive from −6% to +6% strain, which supports G‐FiM ordering (AFM coupling compatible with Fe−O−Os superexchange angles of ≈150°^[^ ^29^, ^30^, ^31^, ^32^ ^]^). C‐AFM ordering emerges at −5% strain, regardless of the positive *J*_2_, because *J*_1_ is large and *J*_3_ and *J*_4_ compete with *J*_2_. Similarly, E‐AFM ordering emerges at +5% strain, regardless of the positive *J*_1_, because *J*_2_ is large and *J*_5_ (23 meV) and *J*_6_ (36 meV), which are large due to strong structural distortions, compete with *J*_1_. Thus, the strong AFM out‐of‐plane NN (*J*_2_) and in‐plane NNN (*J*_5_, *J*_6_) couplings jointly enforce E‐AFM ordering. To compute the critical temperatures, we employ Monte Carlo simulations^[^ ^33^ ^]^ for Ising spins in a 12 × 12 × 12 supercell, using 100 000 sweeps for thermalization and 80 000 additional sweeps in the equilibrium. Without strain we obtain 440 K when only the NN couplings are considered, see Figure S1, Supporting Information, and a substantial reduction to 350 K (due to a slight spin frustration) when both the NN and NNN couplings are included, see **Figure** **4**a,b. The reduced value of 350 K agrees well with the experimental value of 320 K.^[^ ^34^ ^]^ According to Figure 4c, the critical temperature slightly decreases/increases under tensile/compressive strain in the G‐FiM phase, however, always staying above room temperature. For the C‐AFM and E‐AFM phases it is significantly higher due to the large *J*_1_ (C‐AFM phase) and *J*_2_, *J*_5_, and *J*_6_ (E‐AFM phase), respectively, despite competition with *J*_3_ and *J*_4_.

**Figure 5:**
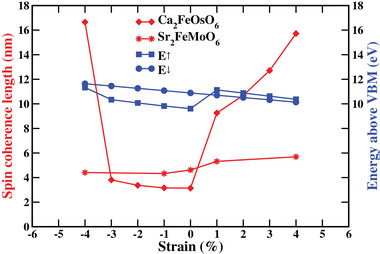
Variation of the spin coherence length and the energies *E* ^↑^ and *E* ^↓^ under strain.

As the net magnetic moment of 2 μ_B_ per formula unit in the G‐FiM phase is of particular interest to room temperature spintronic applications, we estimate the spin coherence length (which determines the size of the spin transfer torque) in the free‐electron model as λ_c_ = π/|*k* ^↑^ − *k* ^↓^|,^[^ ^19^ ^]^ where *k* ^↑^ and *k* ^↓^ refer to the energies *E* ^↑^ and *E* ^↓^, respectively, at which the spin dephasing occurs (Fermi energy or conduction band minimum of the spin channel), implying $\lambda_{c}=\hbar \pi /\sqrt{2m_{e}}\left|\sqrt{E^{↑}}-\sqrt{E^{↓}}\right|$. The values of *E* ^↑^ and *E* ^↓^ can be extracted from the density of states by considering the valence band maximum of the spin channel as the energy zero. The variation of λ_c_ under strain is depicted in **Figure** **5** and compared to the famous room temperature spintronics material Sr_2_FeMoO_6_.^[^ ^3^ ^]^ Interestingly, we find much larger variations for Ca_2_FeOsO_6_ than for Sr_2_FeMoO_6_, resulting in strongly enhanced values at −4% strain and from +1% to +4% strain. The large change from zero to +1% strain can be attributed to a sharp decrease of the difference between *E* ^↑^ and *E* ^↓^ due to an indirect‐to‐direct band gap transition (see below). For increasing tensile strain this difference continues to decrease and λ_c_ increases accordingly. Similarly, the difference sharply decreases from −3% to −4% strain. The C‐AFM and E‐AFM phases provide zero net magnetic moment but still can be of interest to spintronic applications due to their infinite spin coherence length.^[^ ^18^, ^35^ ^]^

**Figure 6:**
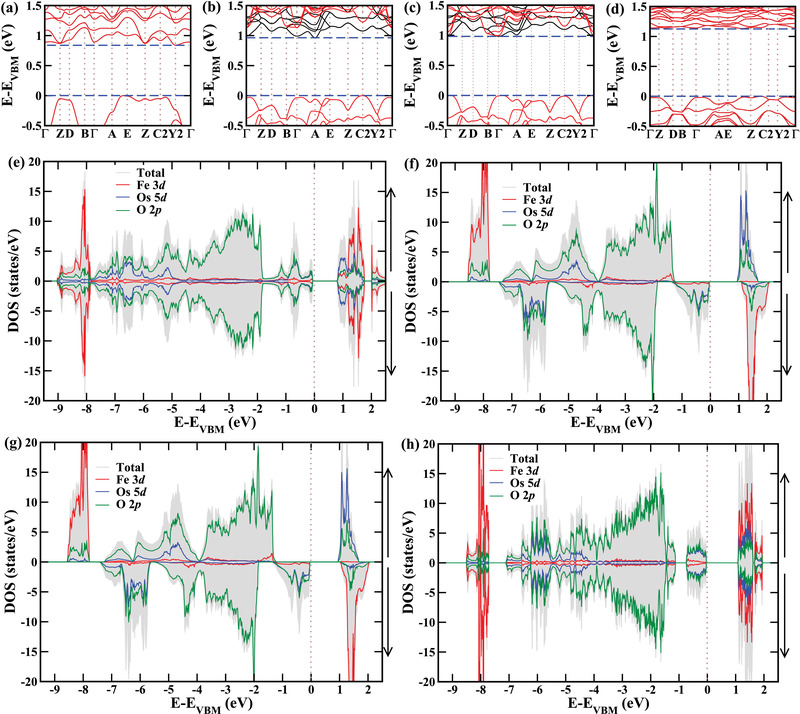
Electronic band structures and densities of states of the a,e) C‐AFM phase at −6% strain (indirect band gap), b,f) G‐FiM phase without strain (indirect band gap), c,g) G‐FiM phase at +1% strain (direct band gap) and, d,h) E‐AFM phase at +6% strain (indirect band gap). As the simulation cell of the E‐AFM phase contains four instead of two formula units, the values of the densities of states are divided by two in this case.

**Figure** **6**a–d shows the electronic band structures of the C‐AFM phase at −6% strain, G‐FiM phase at zero and +1% strain, and E‐AFM phase at +6% strain. While the C‐AFM phase features an indirect band gap, the G‐FiM phase undergoes an indirect‐to‐direct band gap transition between zero and +1% strain. The E‐AFM phase then features again an indirect band gap. In agreement with the zero net magnetic moments, the densities of states of the spin‐majority and spin‐minority channels are identical in the C‐AFM and E‐AFM phases, see Figure 6e,h. In all the phases the Fe and Os atoms maintain 3+ ($t_{2g}^{3}e_{g}^{2}$) and 5+ ($t_{2g}^{3}e_{g}^{0}$) oxidation states, respectively, with high spin configurations. In the G‐FiM phase all three valence electrons of Os belong to the spin‐minority channel and all five valence electrons of Fe belong to the spin‐majority channel, see Figure 6f,g, which reflects AFM superexchange between NN Fe and Os atoms in both the in‐plane and out‐of‐plane directions. The result is a net magnetic moment of 2 μ_B_ per formula unit. In the direct band gap range of the G‐FiM phase (+1% to +4% strain) the spin‐majority channel shows a wide band gap (2.50 eV at +1% strain) and the spin‐minority channel a narrow band gap (1.17 eV at +1% strain). The valence band edge of the spin‐minority channel is dominated by hybridized Os 5d and O 2p states, whereas the conduction band edge is almost entirely due to Fe 3d states. As a consequence, photoexcitation will lead to spatial electron–hole separation between the Fe−Os and Os−Os sublattices, similar to Bi_2_FeCrO_6_.^[^ ^6^ ^]^ As the transition matrix element vanishes due to the intermediate O atom, the electron–hole recombination is suppressed, which is desirable in photovoltaics. The same applies to the E‐AFM phase. While the band gap slightly decreases for increasing tensile strain in the G‐FiM phase (1.17 eV at +1% strain, 1.11 eV at +2% strain, 1.05 eV at +3% strain, 0.98 eV at +4% strain), it increases in the E‐AFM phase (1.11 eV at +5% strain, 1.18 eV at +6% strain).

**Figure 7:**
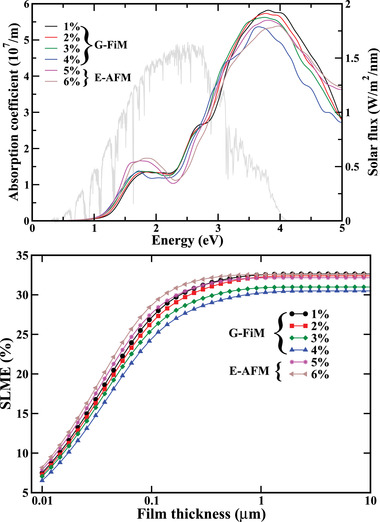
Absorption spectra (the gray curve is the AM 1.5G solar flux) and SLMEs under tensile strain.

To determine the potential of Ca_2_FeOsO_6_ as light absorber in solar energy harvesting, we calculate the absorption spectra, see **Figure** **7**, which show under increasing tensile strain a red‐shift for the G‐FiM phase and a blue‐shift for the E‐AFM phase. Interestingly, the absorption in the energy range from 1.5 to 2.1 eV is stronger in the E‐AFM than in the G‐FiM phase due to the larger density of states near the valence band edge, see Figure 6b,d. To quantify the power conversion efficiency for solar cell applications, we calculate the SLME, which depends on α(ω), the nature of the band gap, and the thickness of the light absorber.^[^ ^36^, ^37^ ^]^ This methodology is known for its accuracy, for example, predicting only slightly overestimated power conversion efficiencies of 26.7% for MAPbI_3_ (MA = CH_3_NH_3_) and 26.4% for FAPbI_3_ (FA = CH(NH_2_)_2_) as compared to the experimental values of 22.1% and 20.2%, respectively.^[^ ^38^ ^]^ According to Figure 7, the obtained SLME values of the G‐FiM phase are highest at +1% strain and those of the E‐AFM phase are highest at +6% strain, which can be attributed to the trends of the band gap discussed before. It turns out that Ca_2_FeOsO_6_ outperforms even the prototypical hybrid perovskite solar cell material MAPbI_3_ (MA = methylammonium; 31% at 1 μm, for example) and, importantly, it is possible to achieve a very high SLME with a thin light absorber. For example, the values of Figure 7 at 0.3 μm significantly exceed the value of 15.4% reported for CH_3_NH_3_Pb(I/Cl)_3_.^[^ ^39^ ^]^

## 4 Conclusions

First‐principles calculations show that the double perovskite Ca_2_FeOsO_6_ undergoes transitions from a G‐FiM phase into a C‐AFM phase under −5% strain (compressive) and into an E‐AFM phase under +5% strain (tensile). Monte Carlo simulations demonstrate that spin frustration reduces the critical temperature of the G‐FiM ordering to reconcile a major discrepancy between experiment and theory. It turns out that the critical temperature remains above room temperature throughout the considered range of epitaxial strain (−6% to +6%). The spin coherence length is found to significantly exceed that of the famous spintronic material Sr_2_FeMoO_6_ under tensile strain, indicating excellent application potential of Ca_2_FeOsO_6_ in spintronics. In addition, the discovered indirect‐to‐direct band gap transition from zero to +1% strain combined with electron–hole separation between the Fe and Os sublattices is interesting for photovoltaics. Ca_2_FeOsO_6_ is found to outperform even the prototypical hybrid perovskite solar cell material MAPbI_3_ in terms of the SLME. The fact that combination of room temperature magnetism with a long spin coherence length and a high SLME is unique in the family of 3d–5d double perovskites highlights the potential of epitaxial strain in tuning the functionalities.

## Conflict of Interest

The authors declare no conflict of interest.

## Supporting information

Supporting Information

## Data Availability

The data that support the findings of this study are available from the corresponding author upon reasonable request.
