# Supplementary material for: Large Spin Coherence Length and High Photovoltaic Efficiency of the Room Temperature Ferrimagnet Ca2FeOsO6 by Strain Engineering
Source: Adv Sci (Weinh). 2022 Jul 21;9(26):2106037. doi: 10.1002/advs.202106037 (PMC9475547; doi:10.1002/advs.202106037)
Supplement: Supplementary file 1 — Supporting Information [file ADVS-9-2106037-s001.pdf]

ADVANCED SCIENCE
Open Access

Supporting Information

for *Adv. Sci.*, DOI 10.1002/advs.202106037

Large Spin Coherence Length and High Photovoltaic Efficiency of the Room Temperature Ferrimagnet Ca_2FeOsO_6 by Strain Engineering

*Paresh C. Rout and Udo Schwingenschlögl**

Supplementary Material

Large spin coherence length and high photovoltaic efficiency of the epitaxial strained room temperature ferrimagnetic Ca_2FeOsO_6

Paresh C. Rout and Udo Schwingenschlögl
Physical sciences and Engineering Division (PSE),
King Abdullah University of Science and Technology (KAUST), Thuwal 23955-6900, Saudi Arabia

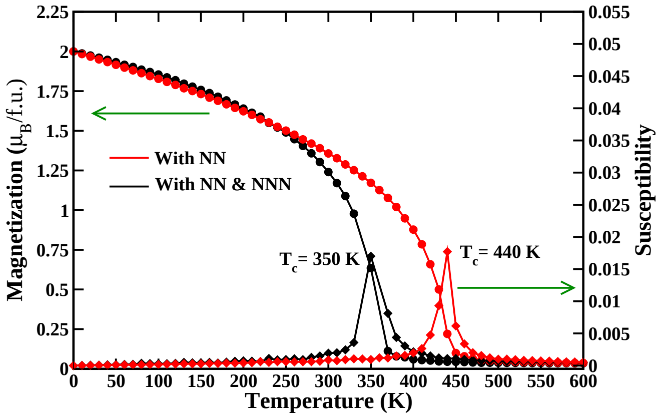

FIG. S1. Temperature dependences of the magnetic susceptibility and total magnetization without strain obtained from classical Monte Carlo simulations, indicating that the critical temperature of the G-FiM phase is 440 K when only the NN couplings are considered and 350 K when both the NN and NNN couplings are considered. The total magnetization rapidly decreases near the critical temperature, indicating a FM-to-paramagnetic phase transition.

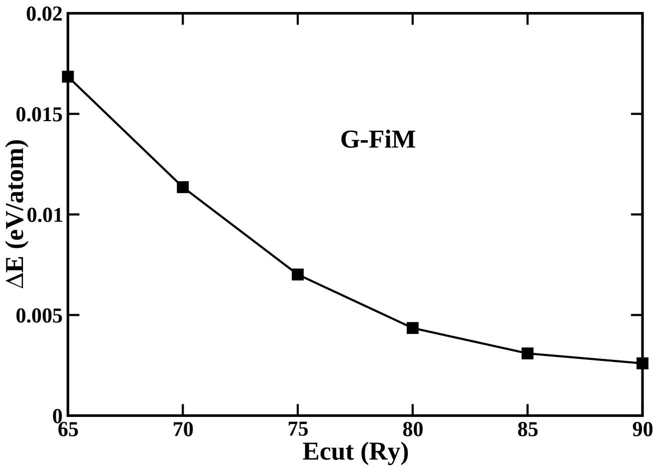

FIG. S2. Total energy convergence with respect to the plane wave cutoff energy.
